# Potential use of COX-2–aromatase inhibitor combinations in breast cancer

**DOI:** 10.1038/sj.bjc.6602690

**Published:** 2005-08-15

**Authors:** N J Bundred, N L P Barnes

**Affiliations:** 1 South Manchester University Hospital, Academic Surgery, Education and Research Centre, Southmoor Road, Manchester M23 9LT, UK

**Keywords:** COX-2, breast cancer, aromatase inhibitors, combination therapy

## Abstract

Cyclooxygenase-2 (COX-2) is overexpressed in several epithelial tumours, including breast cancer. Cyclooxygenase-2-positive tumours tend to be larger, higher grade, node-positive and HER-2/*neu*-positive. High COX-2 expression is associated with poor prognosis. Cyclooxygenase-2 inhibition reduces the incidence of tumours in animal models, inhibits the development of invasive cancer in colorectal cancer and reduces the frequency of polyps in familial adenomatous polyposis (FAP). These effects may be as a result of increased apoptosis, reduced angiogenesis and/or proliferation. Studies of COX-2 inhibitors in breast cancer are underway both alone and in combination with other agents. There is evidence to suggest that combining COX-2 inhibitors with aromatase inhibitors, growth factor receptor blockers, or chemo- or radiotherapy may be particularly effective. Preliminary results from combination therapy with celecoxib and exemestane in postmenopausal women with advanced breast cancer showed that the combination increased the time to recurrence. Up to 80% of ductal carcinomas *in situ* (DCISs) express COX-2, therefore COX-2 inhibition may be of particular use in this situation. Cyclooxygenase-2 expression correlates strongly with expression of HER-2/*neu*. As aromatase inhibitors appear particularly effective in patients with HER-2/*neu*-positive tumours, the combination of aromatase inhibitors and COX-2 inhibitors may be particularly useful in both DCIS and invasive cancer.

Preclinical and clinical evidence shows that prostaglandins play an important role in the growth and development of cancer. Prostaglandin endoperoxide synthase (also called cyclooxygenase; COX) is the rate-limiting enzyme involved in the oxidative transformation of arachidonic acid into prostaglandin H2, which represents the precursor of several bioactive molecules, including prostaglandin E_2_ (PGE_2_), prostacyclin and thromboxane. There are at least two isoforms of the COX enzyme: cyclooxygenase-1 (COX-1) and cyclooxygenase-2 (COX-2) (Hla *et al*, 1999). Both isoforms are found in most human tissues, but while COX-1 is constitutively expressed at low levels (O’Neill and Ford-Hutchinson, 1993), COX-2 expression is an immediate-early response gene that is inducible in response to a variety of stimuli. Cyclooxygenase-2 is induced by a number of promoters (Herschman, 1996), including stimulation of the epidermal growth factor receptor HER-2/*neu* (also called c-erbB2) pathway through RAS and the NF*κ*B and NFIL6 inflammation pathways. Overexpression of COX-2 is detected in several epithelial tumours including those of the colon, lung, breast, prostate, skin, oesophagus, pancreas and bladder. New blood vessels surrounding tumours have COX-2 within them (Sano *et al*, 1995; Chan *et al*, 1999; Tucker *et al*, 1999) and, therefore, COX-2 inhibition targets not only the epithelial cells of tumours but also the endothelial cells. In neoplasia, COX-2 promotes tumour angiogenesis (Chang *et al*, 2004) and inhibits apoptosis (Hsu *et al*, 2000).

Overexpression of COX-2 in the breast tissue of transgenic mice is associated with increased development of breast tumours, but only after multiple pregnancies (Liu *et al*, 2001). Breast tissues that express COX-2 in these transgenic mice have reduced levels of apoptosis compared with normal tissue (Liu *et al*, 2001). In humans, COX-2 is raised in both ductal carcinoma *in situ* (DCIS, Half *et al*, 2002) and invasive breast cancer (Spizzo *et al*, 2003; Boland *et al*, 2004; Figure 1) and appears to be associated with higher proliferation, lower apoptosis and increased new blood vessel formation (Kirkpatrick *et al*, 2001; Boland *et al*, 2004). Indeed, a large study of 1576 patients with invasive breast cancer demonstrated that COX-2 was expressed at high levels in over 50% of patients and these patients had a significantly worse disease-free survival compared with patients whose tumours expressed low-levels of, or absent, COX-2 (Ristimaki *et al*, 2002). Studies with celecoxib, a selective COX-2 inhibitor, in an HER2/*neu*-induced breast cancer model in transgenic mice have revealed that celecoxib reduced the incidence of mammary tumours and lowered mammary PGE_2_ levels by 50% (Howe *et al*, 2002; Lanza-Jacoby *et al*, 2003).

In colorectal cancer and familial adenomatous polyposis (FAP), COX-2 inhibition has been shown to inhibit the development of invasive cancer and reduce the frequency of polyp formation (Giardiello *et al*, 1993; Steinbach *et al*, 2000; Phillips *et al*, 2002; Asano and McLeod, 2004). In a pivotal study, 6 months of treatment with celecoxib, 400 mg b.i.d. (twice the licensed antiarthritis dose) significantly prevented polyp formation compared with placebo (Phillips *et al*, 2002). Studies have also suggested that combinations of COX-2 inhibitors with an epidermal growth factor receptor (EGFR) inhibitor have synergistic benefits in animal models of FAP (min mouse) (Torrance *et al*, 2000). Other studies have shown a potential benefit of COX-2 inhibitors in non-small-cell lung cancer (Altorki *et al*, 2003).

Work from our laboratory (Barnes *et al*, 2003) and others (Masferrer *et al*, 2000; Fu *et al* 2004) has clearly shown that COX-2 inhibition with celecoxib prevents the growth of lung, colon and breast human tumour xenografts in nude mice (Figure 2). The growth of both MDA-MB231 cells, an oestrogen receptor (ER)-negative, EGFR-positive RAS transformed breast cancer cell line and HER18 cells, an ER-positive, HER-2/*neu* transfected MCF-7 breast cancer cell line, were both markedly inhibited by celecoxib in a xenograft model (Figure 2C; Barnes *et al*, 2003). These data suggest that celecoxib may help to prevent ER-negative cancers and be of value in HER-2/*neu*-positive breast cancer. Studies of COX-2 inhibitors in breast cancer are underway as adjuvant therapy.

## MECHANISMS OF ACTION OF COX-2 INHIBITORS

There are three potential anticancer mechanisms for COX-2 inhibition, it may

- inhibit proliferation in epithelial cells,
- increase apoptosis,
- reduce angiogenesis.

### Proliferation

Data from studies that have looked at COX-2 expression and proliferation markers, such as Ki67, have shown a strong correlation between the presence of COX-2 and increased proliferation (Ferrandina *et al*, 2003; Boland *et al*, 2004), but as yet no data have been published to show that inhibition of COX-2 leads to a lower proliferative rate in tumours *in vivo*.

### Apoptosis

Several lines of study have indicated that COX-2 inhibition may increase apoptosis. In a study investigating the effect of celecoxib on prostate cancer cell lines, AKT phosphorylation was significantly inhibited by celecoxib leading to increased caspase-3 activation and apoptosis (Hsu *et al*, 2000). Celecoxib has also recently been shown to decrease the activation of the transcription factor NF*κ*B in lung cancer cell lines (Shishodia *et al*, 2004); celecoxib was found to interfere with the tumour necrosis factor-alpha (TNF-*α*) induced interaction of AKT with IKK (the protein kinase required for NF*κ*B activation). NF*κ*B regulates a wide spectrum of genes including those involved in the immune response, cell proliferation and apoptosis.

Since HER-2/*neu* regulates AKT phosphorylation downstream (Nicholson *et al*, 2003), it is certainly conceivable that inhibiting COX-2 may affect this pathway upstream leading to increased apoptosis. As previously mentioned, in COX-2 transgenic mice, mouse mammary tissue had significantly less apoptosis than normal tissue and had a reduced expression of Bcl_2_-XL and BAX (Liu *et al*, 2001), which are both proapoptotic proteins. Also, it may be that COX-2 affects the mitochondrial pathway (an intrinsic pathway that induces apoptosis in the normal breast).

### Angiogenesis

Celecoxib inhibition of COX-2 is one of the most potent antiangiogenic mechanisms currently available to the clinician. Multiple publications from Dubois and co-workers have indicated that COX-2 inhibition leads to a reduced secretion of vascular endothelial growth factor (VEGF) and *in vitro* reduced endothelial tube formation in matrigel (Tsujii *et al*, 1998; Sawaoka *et al*, 1999; Woods *et al*, 2003). Celecoxib has been shown to inhibit basic fibroblast growth factor-induced angiogenesis *in vivo* using a rat corneal angiogenesis model (Masferrer *et al*, 2000). Furthermore, lung cancer xenografts showed decreased vascularity when implanted into COX-2 null mice (Williams *et al*, 2000). Chang *et al* have shown that COX-2 regulates angiogenesis in normal mammary tissue via PgE_2_ production; therefore, inhibition of angiogenesis by COX-2 inhibitors has the potential for chemoprevention of breast cancer.

In invasive breast cancer, COX-2 expression has been shown to correlate with the levels of angiogenesis (measured by CD-31 staining) in tumours (Davies *et al*, 2003). In addition, preliminary results, from a randomised trial using celecoxib given for 2 weeks before surgery compared with no treatment, have shown a significant fall in serum VEGF levels after 14 days of treatment, indicating that celecoxib may be useful in preventing angiogenesis and lymphovascular spread at around the time of surgery (O-Donoghue *et al*, 2004). Therefore, there is increasing interest in examining COX-2 inhibitors in breast cancer, alone or in combination with other agents, to determine the overall effect on prevention, recurrence after early breast cancer treatment and induction of endocrine response in advanced disease. The antiangiogenic and proapoptotic effects seen indicate that the drug may potentially be effective in both ER-positive and -negative tumours.

## INTEGRATING COX-2 INHIBITION INTO BREAST CANCER TREATMENT

With preclinical studies demonstrating an effect of COX-2 inhibition on tumour growth in animal models and COX-2 being involved in breast carcinogenesis, COX-2 inhibition is being considered for inclusion into breast cancer therapy. It is likely that COX-2 inhibitors will be used as combination therapy with hormonal agents, such as an aromatase inhibitor to decrease aromatisation and angiogenesis, or with growth factor receptor blockers, such as trastuzumab (Herceptin®), to synergise blockade of the HER-2/*neu* RAS pathway inhibition. Also, COX-2 inhibition has been shown in animal models to make tumours significantly more chemo- and radio-sensitive. Therefore, several combinations are being explored in current clinical trials.

Rofecoxib was recently withdrawn from the market due to an increased risk of cardiovascular events found in both the Vioxx Gastrointestinal Outcomes Research (VIGOR) study and the recent Adenomatous Polyp Prevention on Vioxx (APPROVe) trial. The cardiovascular safety of celecoxib is currently being examined following results from one trial, the Adenoma Prevention with Celecoxib (APC) trial, which found patients taking 400 and 800 mg day^−1^ of celecoxib had a 2.5- to 3.4-fold increased risk of major fatal or nonfatal cardiovascular events *vs* placebo (average duration of treatment 33 months). The use of celecoxib in this trial has now been suspended. Data suggest that any cardiovascular concerns may be related to long-term use (>12 months) of celecoxib. By contrast, no increased risk has been seen for celecoxib 400 mg day^−1^ *vs* placebo in two separate long-term studies, the Prevention of Spontaneous Adenomatopus Polyps (PreSAP) trial and the Alzheimer's Disease Anti-inflammatory Prevention Trial (ADAPT). Additionally, no cardiovascular concerns have been noted in over 40 000 celecoxib-treated patients. Several trials investigating celecoxib in preinvasive, invasive and metastatic breast cancer are ongoing as shown in Table 1.

It is recommended that patients with a history of ischaemic heart disease or cardiovascular disease should not receive a COX-2 inhibitor.

### Combining COX-2 inhibitors with aromatase inhibitors

Brueggemeier *et al* (1999, 2001) looked at the levels of aromatase (*cyp19*) and *cox-2* gene expression in breast tissue using the semiquantative, reverse transcriptase polymerase chain reaction (RT-PCR) technique. High levels of *cox-2* mRNA expression led to increased levels of PGE_2_, which in turn increased *cyp19* expression. This was achieved through increased intracellular cAMP levels and activation of the *cyp19* promoter 2, resulting in increased aromatase activity (Richards *et al*, 2002). Zhao *et al* (1996) have shown that the level of aromatase activity is markedly increased in the presence of PGE_2_. Other workers have indicated that the PGE_2_ and cytokines such as interleukin-6 or TNF-*α* regulate aromatase activity in tumour cells (Michael *et al*, 1997; Brueggemeier *et al*, 2001). Therefore, inhibition of PGE_2_ by COX-2 inhibitors may inhibit aromatase activity and, when combined with aromatase inhibitors, reduce tumour recurrence by inhibiting a common target: aromatase.

**Figure 3:**
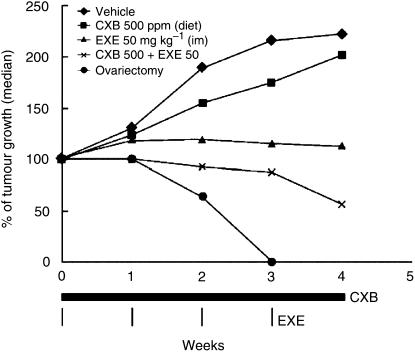
Combined celecoxib (CXB) and exemestane (EXE), reduces mammary tumour growth in a rodent model more effectively than either therapy alone (Pesenti *et al*, 2001).

Pesenti *et al* (2001) provided preclinical data from a rodent model in which celecoxib combined with exemestane significantly inhibited the growth of mammary tumours compared with vehicle or celecoxib alone and slowed the growth of established tumours at 5 weeks (Figure 3). Results of a small, randomised, phase II study in postmenopausal women (*n*=111) with advanced breast cancer treated with exemestane, 25 mg q.d. and celecoxib, 400 mg p.o. b.i.d. indicated a longer time to breast cancer recurrence with no additional side effects from the use of celecoxib and exemestane (Dirix *et al*, 2003). Pharmacogenomic studies are planned to determine the mechanism of action of increased inhibition with celecoxib.

### Other potential COX-2 combinations

#### Trastuzumab

Several studies have shown that up to 80% of DCISs express COX-2 and that its expression correlates strongly with expression of HER-2/*neu* in the same tumours (Half *et al*, 2002; Tan *et al*, 2004). Cyclooxygenase-2 expression has been associated with higher grade ER-negative, HER-2/*neu*-positive tumours and COX-2-positive tumours have been linked with a significantly worse overall survival (Ristimaki *et al*, 2002). Therefore, in the group of women with HER-2/*neu*-positive tumours, it may be beneficial to use COX-2 inhibition in combination with an agent such as trastuzumab (Herceptin®), which blocks the extracellular domain of the HER-2/*neu* receptor and inhibits RAS and MAPkinase signalling. A phase II, randomised trial of trastuzumab, with or without celecoxib, in a series of 12 patients with metastatic breast cancer who had previously progressed after trastuzumab-based treatments, found that there was no treatment effect, although the drug combination was well tolerated (Dang *et al*, 2004). This study consisted only of patients who had been pretreated with trastuzumab. The effect on treatment-naïve patients is still unknown and being investigated in an ongoing trial.

#### Chemotherapy

Blocking PGE_2_ production by inhibiting COX-2 has recently been shown to have effects upstream of the HER-2/*neu* pathway resulting in decreased HER-2/*neu* protein levels and increased sensitivity of cancer cells to chemotherapeutic treatment (Benoit *et al*, 2004). Coadministration of celecoxib and cyclophosphamide was significantly more effective in preventing growth of Lewis lung cancer tumours than either drug alone (Koki *et al*, 1999) and studies of combination of several chemotherapy agents with celecoxib have indicated that celecoxib lowers the threshold of sensitivity to chemotherapy (Altorki *et al*, 2003; Nakata *et al*, 2004). In Europe, a phase III, multicentre, double-blind, randomised trial of celecoxib *vs* placebo following chemotherapy (REACT Trial; Current Controlled Trials) has been initiated in primary breast cancer patients, although the protocol for this study is currently under review. Primary breast cancer patients who have completed surgery, neoadjuvant chemotherapy and radiotherapy are randomised to receive celecoxib, 400 mg twice a day for 2 years, or placebo, with exemestane given to all ER-positive patients for 5 years. This study aims to determine whether the addition of celecoxib improves overall survival in patients at high risk of recurrence.

### COX-2 and aromatase inhibition in DCIS

The high expression of HER-2/*neu* and COX-2 in DCIS (particularly high grade) combined with the clear correlation between proliferation and COX-2 expression in DCIS (Boland *et al*, 2004) has stimulated interest in the use of COX-2 inhibitors to prevent recurrent DCIS in ER-positive and -negative disease. Clear evidence from both the ATAC (Baum *et al*, 2003) study and the Letrozole/Tamoxifen Neoadjuvant Trial has suggested that aromatase inhibition is the treatment of choice in HER-2/*neu*-positive, ER-positive tumours (Ellis *et al*, 2001). In a 3-month neoadjuvant study, letrozole was 28 times more likely than tamoxifen to induce response in HER-2/*neu*-positive, ER-positive, invasive breast cancer, with 88% of patients responding to the aromatase inhibitor and 21% to tamoxifen. By contrast, in HER-2/*neu*-negative, ER-positive patients, 54% responded to letrozole and 42% to tamoxifen (Ellis *et al*, 2001). The recently published results of the ATAC trial comparing anastrozole with tamoxifen demonstrated a reduction in recurrence of over 8% in ER-positive, PR-negative patients (predominantly HER-2/*neu*-positive group) compared with a 4% difference in the ER-positive, PR-positive group (Baum *et al*, 2003; Dowsett *et al*, 2003). This would suggest that aromatase inhibition would be more beneficial in those patients who express HER-2/*neu*. The mechanism for this is not fully clear; however, tamoxifen translocates the ER complex to the nucleus, opening up DNA and allowing some agonist activity with coactivators, whereas aromatase inhibition does not (Ellis *et al*, 2001). Therefore, the HER-2/*neu* pathway cannot affect the coactivators or corepressors that have been induced by translocating the ER to the nucleus. There is evidence that expression of AIB (amplified in breast cancer 1), a nuclear coactivator complex, is upregulated in HER-2/*neu*-positive tumours and is associated with endocrine resistance (Osborne *et al*, 2002). Preventing translocation of the ER to the nucleus lowers oestrogen levels in the breast and tumour tissue and lowers AIB levels, therefore decreasing endocrine resistance (Shou *et al*, 2002).

With 70% of DCISs expressing HER-2/*neu* (Tan *et al*, 2002), it is possible that combining COX-2 inhibition with an aromatase inhibitor in DCIS patients will reduce recurrence in both the ipsilateral and contralateral breast. Early, placebo-controlled studies in DCIS are assessing the effects of administering celecoxib for 14 days on proliferation, apoptosis and angiogenesis in the presurgical setting. Studies in the USA are looking at the effect of celecoxib on ER-negative DCIS in this setting and in the UK on ER-positive DCIS. In addition, trials have been proposed using adjuvant celecoxib in ER-negative DCIS to determine whether it prevents recurrence after wide local excision. Given the lack of chemopreventive agents that can be used to prevent ER-negative breast cancer or used as adjuvant therapy in ER-negative breast cancer, there is a potential primary role for celecoxib in this setting.

## CONCLUSIONS

Animal studies and early patient trials have shown encouraging results for the use of COX-2 inhibition alone and/or with other therapeutic agents in breast cancer and other oncology settings, including colorectal cancer and non-small-cell lung cancer. In particular, combining an aromatase inhibitor with a COX-2 inhibitor has been hypothesised to reduce overall disease recurrence by inhibiting a common target, aromatase. A number of trials are ongoing to test this hypothesis further and results are awaited with interest.

## Figures and Tables

**Figure 1:**
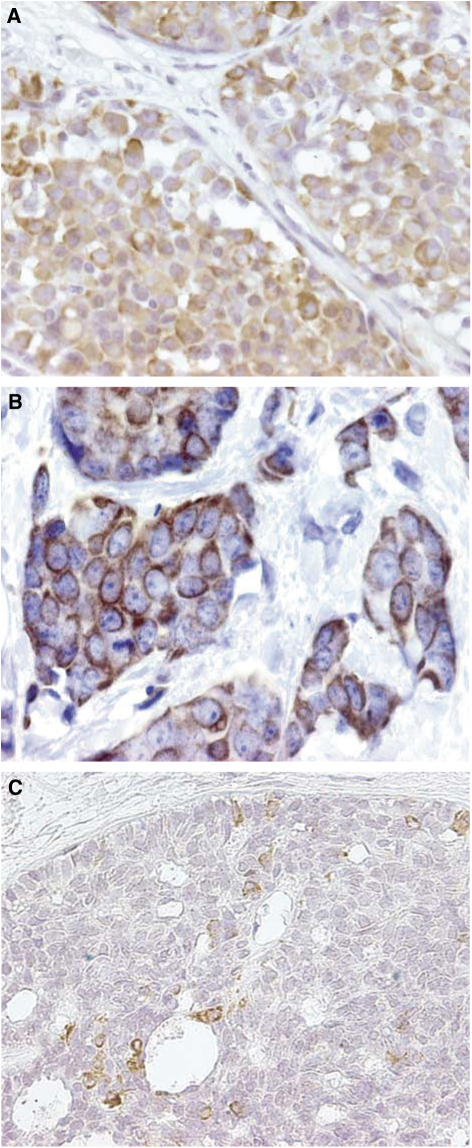
COX-2 is strongly expressed in the cell cytoplasm in (**A**) ductal carcinoma *in situ* and (**B**) invasive breast cancer. For comparison, (**C**) shows COX-2 as negatively expressed in a ductal carcinoma *in situ* with little staining.

**Figure 2:**
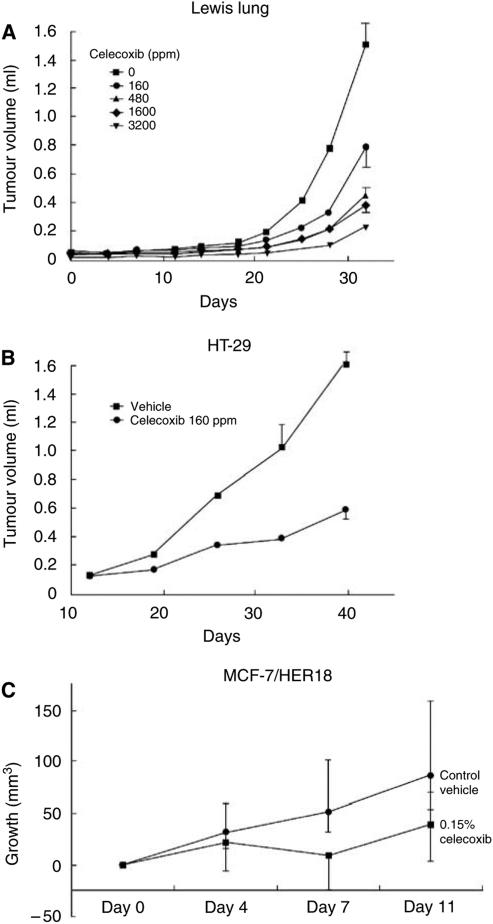
Celecoxib, a COX-2 inhibitor, slows growth of cancer cell lines derived from (**A**) lung, (**B**) colon and (**C**) breast when xenografted into mice (Masferrer *et al*, 2000; Barnes *et al*, 2003). (**C**) Median tumour growth, bars=interquartile range. Figures (**A**) and (**B**) reproduced with permission from Masferrer *et al* (2000).

**Table 1:** Overview of current clinical trials of COX-2 inhibitors in the treatment of breast cancer

**Trial**	**Principal investigator (Ref)**
UK multicentre phase III trial of exemestane and COX-2 inhibition in ER-positive DCIS	Bundred (National Cancer Research Network, 2005)
Neoadjuvant celecoxib plus fluorouracil, epirubicin and cyclophosphamide for the treatment of locally advanced breast cancer	Chow *et al* (2003)
KUMC-HSC-8919-02: phase II chemoprevention study of celecoxib in premenopausal women at high risk of ER-negative breast cancer	Fabian (National Cancer Institute, 2005a)
Italian breast cancer trial of celecoxib in combination with weekly taxotere and capecitabine as first-line therapy in advanced breast cancer	Gasparini *et al* (2003)
ICCG: pilot study, DNA microarray analysis of human breast cancer before and after treatment with COX-2 inhibitors: search for biomarkers	Hupperets, Wagstaff (Gasparini *et al*, 2003)
Royal Infirmary phase III, randomised, placebo-controlled trial of celecoxib in patients with metastatic breast cancer	McMillan (Gasparini *et al*, 2003)
Phase 1 trial of vinorelbine and celecoxib in treating women with relapsed or metastatic breast cancer	Overmoyer (Clinical Trials.gov)
MSKCC-03027: phase I, randomised study of celecoxib in postmenopausal women with invasive breast cancer undergoing surgery, to look for suppression of aromatase activity and biomarkers of effect	Port, Hudis (National Cancer Institute, 2005b)
CALGB-40105: phase II, randomised study of celecoxib in women with metastatic or recurrent breast cancer, comparing low dose to high-dose treatment. Further recruitment of patients to celecoxib is under discussion.	Shapiro (National Cancer Institute, 2005c)
NCI-04-C-0044: phase II study of exemestane alone or in combination with celecoxib in postmenopausal women at high risk for invasive breast cancer	Zujewski (National Cancer Institute, 2005d)

DCIS=ductal carcinoma *in situ*; ER=oestrogen receptor.
